# Service Quality and Patient Satisfaction of Internet Hospitals in China: Cross-Sectional Evaluation With the Service Quality Questionnaire

**DOI:** 10.2196/55140

**Published:** 2024-11-08

**Authors:** Tao Han, Qinpeng Wei, Ruike Wang, Yijin Cai, Hongyi Zhu, Jiani Chen, Zhiruo Zhang, Sisi Li

**Affiliations:** 1 School of Public Health Shanghai Jiao Tong University School of Medicine Shanghai China

**Keywords:** service quality, SERVQUAL, Service Quality Questionnaire, internet hospital, e-hospital, digital medical care, health care professionals, Chinese digital health care

## Abstract

**Background:**

Internet hospitals, which refer to service platforms that integrate consultation, prescription, payment, and drug delivery based on hospital entities, have been developing at a rapid pace in China since 2014. However, assessments regarding their service quality and patient satisfaction have not been well developed. There is an urgent need to comprehensively evaluate and improve the service quality of internet hospitals.

**Objective:**

This study aims to investigate the current status of patients’ use of internet hospitals, as well as familiarity and willingness to use internet hospitals, to evaluate patients’ expected and perceived service qualities of internet hospitals using the Chinese version of the Service Quality Questionnaire (SERVQUAL-C) with a national representative sample, and to explore the association between service quality of internet hospitals and patients’ overall satisfaction toward associated medical platforms.

**Methods:**

This cross-sectional survey was conducted through face-to-face or digital interviews from June to September 2022. A total of 1481 outpatient participants (635 men and 846 women; mean age 33.22, SD 13.22). Participants reported their use of internet hospitals, and then rated their expectations and perceptions of service quality toward internet hospitals via the SERVQUAL-C, along with their demographic information.

**Results:**

Among the surveyed participants, 51.2% (n=758) of participants had used internet hospital service or services. Use varied across age, education level, and annual income. Although the majority of them (n=826, 55.8%) did not know internet hospital services well, 68.1% (n=1009) of participants expressed the willingness to adopt this service. Service quality evaluation revealed that the perceived service quality did not match with the expectation, especially the responsiveness dimension. Important-performance analysis results further alerted that reliable diagnosis, prompt response, clear feedback pathway, and active feedback handling were typically the services awaiting substantial improvement. More importantly, multiple linear regressions revealed that familiarity and willingness to use internet hospital services were significant predictors of satisfaction, above and over tangibles, reliability, and empathy service perspectives, and demographic characteristics such as gender, age, education level, and annual income.

**Conclusions:**

In the future, internet hospitals should focus more on how to narrow the gaps between the expected and perceived service quality. Promotion of internet hospitals should also be facilitated to increase patients’ familiarity with and willingness to use these services.

## Introduction

### Internet Hospital Services in China

Internet hospital (or e-hospital) services have been developing at a rapid pace in China since 2014, which refer to hospital-based service platforms that integrate consultation, prescription, payment, and drug delivery, mostly featured in digital follow-up and routine consultations [1,2]. To date, China has witnessed the establishment of over 1700 internet hospitals with an extensive array of services [3]. The rapid growth of internet hospitals in China is expected to enhance the availability and accessibility of medical resources nationwide while controlling the costs associated with distant medical services [4]. Additionally, the strike of the COVID-19 pandemic has further expedited the advancement and acceptance of remote medical care, with internet hospitals playing a pivotal role [5,6]. However, despite the rapid development of internet hospitals, assessments regarding their service quality and patient satisfaction have not been well developed [7]. This study attempted to fill in this research gap.

Internet hospital services heavily rely on various digital platforms such as smartphone apps or miniprograms integrated into popular social media platforms (eg, WeChat or Alipay). The service quality provided by internet hospital platforms directly impacts the medical experience of patients as it encompasses (1) appointment, registration, guided triage, and referral during the previsit stage; (2) remote payment, and instant access to case and test reports during the visit stage; and (3) prescription management, drug delivery, health monitoring, and remote health education during the postvisit stage. Granted these benefits, it was undeniable that the rapid development and engagement of internet hospitals had raised several practical issues such as limited service accessibility to older people and risks of personal information leakage. Therefore, it was critical to comprehensively assess, and therefore, improve the service quality of internet hospitals [8].

### The Service Quality Questionnaire

The service quality model was initially developed by Parasuraman et al [9] to investigate potential causes of service quality issues and assist managers in improving service quality. The model was further refined and a measurement tool was developed accordingly, namely, the Service Quality Questionnaire (SERVQUAL) [10,11], which measures customers’ expectations and perceptions of the service quality provided by an entity, reflecting their attitudes toward the service provider [10], highlighting the gaps between what customers believe should be provided (expectations) and what they believe they have received (perceptions). The SERVQUAL questionnaire consists of 5 dimensions (22 items) [10]: tangibles, which refers to the physical facilities, equipment, and appearance of personnel; reliability, which assesses the ability to deliver services accurately and dependably; responsiveness, which examines the willingness to assist customers and provide prompt service; assurance, which evaluates employees’ knowledge, courtesy, and ability to convey trust and confidence; and empathy, which assesses the level of care and individualized attention provided to customers. The SERVQUAL questionnaire has been applied to evaluate service quality in health-related contexts [12-14], demonstrating its suitability for assessing medical service quality. Therefore, it was adopted to evaluate the service quality of Chinese internet hospitals and to identify the possible gaps between expected and perceived service qualities.

### This Study

To briefly summarize, the purpose of this study was threefold, (1) to investigate the current status of Chinese patients’ use of internet hospitals, as well as their familiarity and willingness to use internet hospitals; (2) to evaluate Chinese patients’ expected and perceived service qualities of internet hospitals using SERVQUAL with a nationally representative sample; and (3) to explore the association between service quality of internet hospitals and overall satisfaction of associated medical platforms.

## Methods

### Participants

A total of 1586 participants were recruited via convenient sampling from 31 provinces in China. Inclusion criteria were (1) had used medical services in China within 1 year and (2) consented to participate in this study.

### Procedure

A cross-sectional e-survey was administered on the SoJump platform (a popular Chinese digital survey platform) from June to September 2022. Recruitment for the survey was carried out through WeChat, a widely used social media platform in China. Potential participants were invited to complete the survey via WeChat moments. Investigators received digital data collection training sessions in advance for survey distribution, data collection monitoring, and quality control. Research team members conducted quality control on submitted responses based on (1) duplicate IP addresses, (2) shorter-than-expected response time window, and (3) incomplete responses. A total of 105 responses that failed at these criteria were excluded from the following analyses. Therefore, the final sample consisted of 1481 participants. Additionally, the 758 people who self-reported having used internet hospital services were further included in the analysis.

### Ethical Considerations

This study was approved by the Shanghai Jiao Tong University School of Medicine Public Health and Nursing Research Ethics Committee (reference: SJUPN-202203). All eligible participants who consented to participate were informed about the study’s purpose and procedure and then were asked to respond to the e-survey without interference. Data collection was conducted anonymously to ensure confidentiality. For participants deemed minor according to their reported age, further consent was requested from their legal guardians. Participants did not receive any material or monetary rewards for participation.

### Materials

Participants responded to a battery of surveys including SERVQUAL-C, satisfaction with internet hospital services, and their demographic information.

#### Internet Hospital Use and Experience

Patients first reported whether they had used the internet hospital (0=no or 1=yes), together with the frequency of use: “How often do you use the internet hospital” (1=never used before to 5=more than five times per year). Reasons for and against using internet hospitals were followed via multiple-choice questions (ie, “What are your reasons for using internet hospitals?” and “What are your reasons against using internet hospitals?”). Then, participants reported their overall familiarity, willingness to use, and satisfaction with internet hospitals by answering the questions “How much do you know about internet hospitals?” (1=barely to 5=very well), “How willing are you to use internet hospitals?” (1=very unwilling to 5=very willing), and “All things considered, how satisfied were you toward Chinese internet hospitals in general?” (1=not at all satisfied to 10=extremely satisfied).

#### Service Quality of Internet Hospitals

The 22-item SERVQUAL questionnaire consists of five dimensions of service quality (4 to 5 items each; 1=very poor to 5=very good), namely tangibles (TG; eg, “The aesthetic degree of internet hospital platform page design”), reliability (RA; eg, “Reliability of treatment results in internet hospitals”), responsiveness (RS; eg, “The timeliness of the internet hospital doctor’s response”), assurance (AS; eg, “The extent to which the internet hospital platform meets the needs of patients”), and empathy (EP; eg, “The extent to which internet hospitals provide personalized care for patients”), each question measures both the perception and the expectation perspectives, respectively. Therefore, SERVQUAL yields three scores, specifically, the perception (P) and the expectation (E) scores, which are computed by averaging the 22 items from each perspective, respectively, and the gap score, which is further computed by subtracting the latter from the former (ie, P–E).

#### Sociodemographic Information

At the end of the questionnaire, participants reported their sociodemographic information, including gender (0=man and 1=woman), age (1=19 years or younger, 2=20-29 years, 3=30-39 years, 4=40-49 years, and 5=50 years or older), location (1=eastern region, 2=central region, 3=western region, and 4=northeast region), marital status (1=unmarried and 2=married), education level (1=below high school’s degree, 2=high school’s degree, 3=bachelor’s degree, and 4=higher than bachelor’s degree), as well as personal annual income (1=¥50,000 or below, 2=¥50,001-100,000, 3=¥100,001-150,000, 4=¥150,001-200,000, and 5=¥200,001 or above). The exchange rate of US $ to RMB ¥ mentioned in this paper is 1:7.0354.

### Statistical Analysis

All personal identifiers have been removed prior to data analysis to ensure anonymization. All data were analyzed with SPSS (version 25.0; IBM Corp) and R (version 4.2.0; R Core Team). The questionnaire was calculated to reflect internet hospital patients’ evaluations. Additionally, important-performance analysis (IPA) [15] was used to compare patients’ expectations and perceptions of the services provided by internet hospitals and to reveal critical management perspectives. Finally, multiple linear regressions were also conducted to examine the association between service quality, demographics, familiarity, and willingness in patient satisfaction. All continuous variables have been centralized.

## Results

Table 1 presents the sociodemographic characteristics of all the participants (n=1481; 635 men and 846 women). Participants were mainly women (n=846, 57.1%), aged between 20 and 29 (n=429, 29%), and married (n=820, 55.4%). The majority of the sample received a bachelor’s degree education (n=969, 65.4%) and received less than 50,000 Chinese Yuan per year.

It was found that 51.2% (n=758) of patients had used internet hospitals. Results suggested that there were significant differences in whether patients had used the internet hospital at different levels of age (*P*<.001), education level (*P*=.006), and annual income (*P*<.001), while differences in gender or marital status were not statistically significant.

Specifically, patients in their 20s (n=268, 62.5%) and 30s (n=190, 72%) had a higher percentage of using internet hospitals. Moreover, the use of internet hospitals varied among patients with different levels of education and annual income. A total of 59.6% (n=65) of patients with higher than bachelor’s degrees had tried internet hospitals, compared with 40% (n=145) of those with lower than high school degrees. Only 39.9% (n=265) of the patients with an annual income of below ¥50,000 had participated in internet hospitals.

A total of 51.2% (n=758) of the patients had used internet hospitals, among which 42.9% (n=325) of the patients used internet hospitals 1-3 times per year. It was also found that there was diversity in the patients’ familiarity and willingness among those who have ever used internet hospital service before. Patients who knew internet hospitals well or excellently only accounted for 26.9% (n=398) and 17.4% (n=258), respectively. A total of 20.3% (n=301) of the patients had poor (n=230, 15.5%) or terrible (n=71, 4.8%) familiarity with internet hospitals. In terms of willingness, 68.1% (n=1009) of the patients gave positive answers, showing a willingness to use internet hospitals. A total of 29% (n=429) of the patients had neutral willingness, and only 2.9% (n=43) of the patients were unwilling to participate in internet hospitals.

**Table 1:** Sociodemographic characteristics and use of internet hospital service among Chinese patients from June to September 2022 (n=1481).

		Use of internet hospital service				*P* value
		Overall, n (%)	Yes, n (%)	No, n (%)		
**Gender**						.56
	Man	635 (42.9)	331 (43.7)	304 (42)		
	Woman	846 (57.1)	427 (56.3)	419 (58)		
**Age (in years)**						<.001
	≤19	274 (18.5)	97 (12.8)	177 (24.5)		
	20*-*29	429 (29)	268 (35.4)	161 (22.3)		
	30*-*39	264 (17.8)	190 (25.1)	74 (10.2)		
	40*-*49	332 (22.4)	130 (17.2)	202 (27.9)		
	≥50	182 (12.3)	73 (9.6)	109 (15.1)		
**Location**						<.001
	Eastern Region	906 (61)	476 (62.8)	427 (59.1)		
	Central Region	272 (18.4)	128 (16.9)	144 (19.9)		
	Western Region	264 (17.8)	118 (15.6)	146 (20.2)		
	Northeast Region	42 (2.8)	36 (4.7)	6 (0.8)		
**Marital status**						.31
	Unmarried	661 (44.6)	328 (43.3)	333 (46.1)		
	Married	820 (55.4)	430 (56.7)	390 (53.9)		
**Education level**						.006
	<High school degree	145 (9.8)	58 (7.7)	87 (12)		
	High school degree	258 (17.4)	143 (18.9)	115 (15.9)		
	Bachelor’s degree	969 (65.4)	492 (64.9)	477 (66)		
	>Bachelor’s degree	109 (7.4)	65 (8.6)	44 (6.1)		
**Annual income (US $1=RMB 7.0354)**						<.001
	≤ ¥50,000	664 (44.8)	265 (35)	399 (55.2)		
	¥50,001*-*100,000	353 (23.8)	207 (27.3)	146 (20.2)		
	¥100,001*-*150,000	258 (17.4)	158 (20.8)	100 (13.8)		
	¥150,001*-*200,000	118 (8)	69 (9.1)	49 (6.8)		
	≥ ¥200,001	88 (5.9)	59 (7.8)	29 (4)		

Table 2 presents patients’ reasons for (n=758) and against (n=723) using internet hospitals. Major reasons for and against using internet hospitals were time-saving (n=500, 66%) and inaccuracy in diagnosis or treatment (n=427, 59.1%), respectively. Meanwhile, cost-saving (n=45, 5.9%) and hard-to-find suitable platforms (n=235, 32.5%) were the least concerned.

Table 3 shows the expectation, perception, and gap scores of the SERVQUAL questionnaire. In the evaluation of Chinese internet hospital service, reliability (mean E 4.12, SD 0.67) received the highest score while empathy (mean E 4.06, SD 0.40) received the lowest score for the expectation perspective, and when it comes to perception perspective, assurance (mean P 3.87, SD 0.74) received the highest score while empathy (mean P 3.79, SD 0.80) still received the lowest score. Across all dimensions, the perception did not meet the expectation of service quality of internet hospitals (namely, all gap scores were negative), and it was noteworthy that the largest gap was observed in the responsiveness dimension.

**Figure 1:**
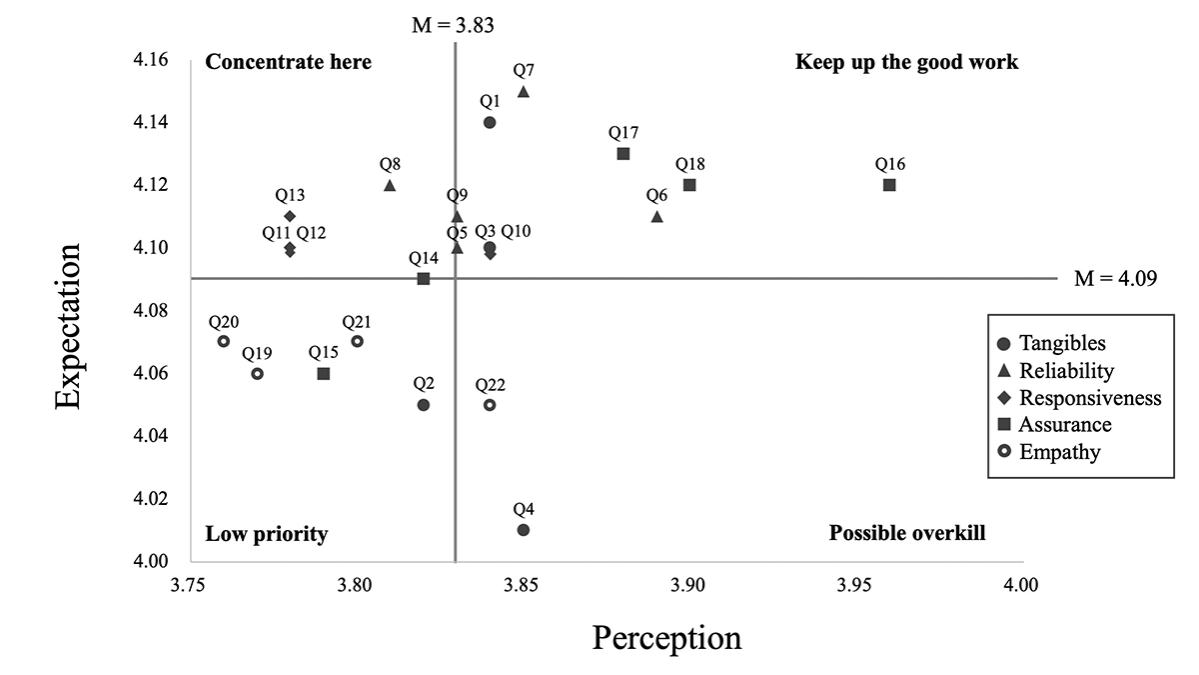
Importance-performance analysis of internet hospital service quality among Chinese patients from June to September 2022 (n=758). Grid lines represent mean scores of expectation and perception, respectively. Solid dots represent tangibles (namely, Q1 to Q4), triangles represent reliability (namely, Q5 to Q9), diamonds represent responsiveness (namely, Q10 to Q13), squares represent assurance (namely, Q14 to Q18), and circles represent empathy (namely, Q19 to Q22).

IPA results are presented in Figure 1. Specifically, the x-axis represents the perception while the y-axis represents the expectation scores, therefore, items in Quadrant I were service features where the performance matched the importance (eg, Q7, protected privacy), while Quadrant II were their counterparts where service qualities should be boosted (eg, Q13, active feedback handling). More importantly, most of the responsiveness features fell into Quadrant II, indicating the perceived responsiveness of internet hospitals in the eyes of patients is less than expected and awaits substantial improvement. Quadrant III consisted of items with low expectations and low perceptions (eg, Q19, personalized care), predominantly the empathy items, where lower expectations led to these items being given a lower priority. Quadrant IV consisted of items with perceptions exceeding expectations, (eg, Q22, reasonable charge), reflecting the strengths and attractiveness of internet hospital service.

Table 4 presents the results of three multiple linear regression models predicting patients’ overall satisfaction with Chinese internet hospitals. Model 1 included five dimensions of internet hospitals’ service quality. In Model 2, two variables regarding familiarity and willingness to use internet hospitals were further added. Finally, Model 3 further included gender, age, location, marital status, education level, and annual income using the “enter” method to examine the associations between service quality perspectives, familiarity, and willingness to use internet hospitals above and beyond these control variables.

Model 1 (adjusted *R*^2^*=*0.526; *F*_5,752_=168.8; *P*<.001) revealed that among the included SERVQUAL-C dimensions, only tangibles (*P*<.001), reliability (*P*=.06), and empathy (*P*<.001) were significant and positively associated with patients’ overall satisfaction while others were not (Bs<.250; *P*s>.066). Among the significant predictors, empathy contributed the most to the explained variance in patient satisfaction.

Model 2 (Δ*R*^2^*=*0.033; Δ*F*_7,750_=29.7; *P*<.001) revealed that familiarity and willingness were significantly and positively associated with patients’ satisfaction, above and beyond the SERVQUAL dimensions, and the inclusion of which further resulted in nonsignificance of the reliability dimension (B=.260; *P*=.08). Willingness appeared a stronger predictor than familiarity in predicting patient’s satisfaction while controlling the SERVQUAL-C dimensions.

**Table 2:** Reasons for and against using internet hospitals among Chinese patients from June to September 2022^a^.

		Count, n (%)
**Reasons** **for** **us** **ing** **internet hospital** **s (n=758)**		
	No queuing could save time	500 (66)
	No need to commute	364 (48)
	Unable or inconvenient to seek offline medical treatment	306 (40.4)
	Simplified medical processes in internet hospitals, easy to operate	319 (42.1)
	Only mild conditions so no need to go for offline consultation	159 (21)
	Access to quality medical resources in more developed areas	126 (16.6)
	Internet hospitals are cost-saving	45 (5.9)
**Reasons against using internet hospitals (n=723)**		
	Inaccuracy in diagnosis or treatment, hard to trust	427 (59.1)
	Delay in medical treatment due to untimely digital response	385 (53.3)
	Internet hospital operation is complicated and inconvenient	290 (40.1)
	Worried about the leakage of private, medical information	275 (38)
	Hard to find suitable internet hospital platforms	235 (32.5)

^a^Responses are presented in descending order in frequency.

**Table 3:** Expectations, perceptions, and gaps in service quality of internet hospitals according to SERVQUAL^a^ among Chinese patients from June to September 2022 (n=758).

Item			Mean (SD)				Gaps
			E^b^		P^c^		
**TG^d^**							
	Q1: smooth operation	4.14 (0.76)		3.84 (0.87)		–0.30	
	Q2: rich functions	4.05 (0.82)		3.82 (0.87)		–0.23	
	Q3: clear layout	4.10 (0.78)		3.84 (0.89)		–0.26	
	Q4: aesthetical layout	4.01 (0.81)		3.85 (0.86)		–0.16	
	Dimension mean	4.08 (0.68)		3.84 (0.76)		–0.24	
**RA^e^**							
	Q5: comprehensive departments	4.10 (0.80)		3.83 (0.88)		–0.27	
	Q6: professional health care	4.11 (0.78)		3.89 (0.85)		–0.22	
	Q7: protected privacy	4.15 (0.81)		3.85 (0.88)		–0.30	
	Q8: reliable diagnosis	4.12 (0.78)		3.81 (0.87)		–0.31	
	Q9: dependable service	4.11 (0.75)		3.83 (0.90)		–0.28	
	Dimension mean	4.12 (0.67)		3.84 (0.73)		–0.28	
**RS^f^**							
	Q10: accurate guidance	4.10 (0.77)		3.84 (0.84)		–0.26	
	Q11: Prompt response	4.10 (0.80)		3.78 (0.97)		–0.32	
	Q12: Clear feedback pathway	4.10 (0.79)		3.78 (0.94)		–0.32	
	Q13: Active feedback handling	4.11 (0.80)		3.78 (0.95)		–0.33	
	Dimension mean	4.10 (0.69)		3.80 (0.81)		–0.30	
**AS^g^**							
	Q14: trustworthiness of the platform	4.09 (0.77)		3.82 (0.88)		–0.27	
	Q15: Satisfaction of the demand	4.06 (0.78)		3.79 (0.89)		–0.27	
	Q16: Attitude of the doctor	4.12 (0.77)		3.96 (0.82)		–0.16	
	Q17: Rationality of the process	4.13 (0.77)		3.88 (0.85)		–0.25	
	Q18: Standardization of the operation	4.12 (0.77)		3.90 (0.86)		–0.22	
	Dimension mean	4.10 (0.67)		3.87 (0.74)		–0.23	
**EP^h^**							
	Q19: personalized care	4.06 (0.82)		3.77 (0.94)		–0.29	
	Q20: understood need	4.07 (0.80)		3.76 (0.89)		–0.31	
	Q21: offered medical advice	4.07 (0.78)		3.80 (0.92)		–0.27	
	Q22: reasonable charge	4.05 (0.80)		3.84 (0.90)		–0.21	
	Dimension mean	4.06 (0.70)		3.79 (0.80)		–0.27	
Overall			4.09 (0.63)		3.83 (0.71)		–0.26

^a^SERVQUAL: Service Quality Questionnaire.

^b^E: Expected service quality estimated by SERVQUAL-C.

^c^P: Perceived service quality estimated by SERVQUAL-C.

^d^TG: Tangibles.

^e^RA: Reliability.

^f^RS: Responsiveness.

^g^AS: Assurance.

^h^EP: Empathy.

**Table 4:** Predictors of patient satisfaction with internet hospital services: service quality, familiarity, willingness, and demographics among Chinese patients from June to September 2022 (n=758).

				B		SE		*P* value		95% CI		*η* ^2^				
**Model 1^a^**																
	Tangibles		.445		0.124		<.001		0.201 to 0.689		0.016					
	Reliability		.373		0.153		.02		0.073 to 0.673		0.008					
	Responsiveness		.250		0.136		.07		–0.016 to 0.516		0.004					
	Assurance		.234		0.160		.15		–0.081 to 0.548		0.003					
	Empathy		.689		0.137		<.001		0.420 to 0.958		0.032					
**Model 2^b^**																
	Tangibles		.359		0.120		.003		0.123 to 0.595		0.012					
	Reliability		.260		0.148		.08		–0.031 to 0.551		0.004					
	Responsiveness		.210		0.132		.11		–0.048 to 0.468		0.003					
	Assurance		.217		0.155		.16		–0.087 to 0.520		0.003					
	Empathy		.636		0.132		<.001		0.376 to 0.896		0.030					
	Familiarity		.167		0.057		.004		0.055 to 0.280		0.011					
	Willingness		.397		0.074		<.001		0.252 to 0.541		0.037					
**Model 3^c^**																
	Tangibles		.347		0.119		.005		0.104 to 0.570		0.011					
	Reliability		.280		0.147		.06		–0.008 to 0.569		0.005					
	Responsiveness		.217		0.130		.09		–0.038 to 0.472		0.004					
	Assurance		.251		0.153		.10		–0.049 to 0.551		0.004					
	Empathy		.575		0.130		<.001		0.319 to 0.831		0.026					
	Familiarity		.131		0.059		.03		0.015 to 0.247		0.007					
	Willingness		.384		0.073		<.001		0.241 to 0.527		0.037					
	Gender		–.233		0.094		.01		–0.417 to –0.049]		0.008					
	**Age (years; reference:** ***≤*** **19** **)**												0.036			
		20-29	.724		0.161		<.001		0.407 to 1.040							
		30-39	.879		0.183		<.001		0.520 to 1.238							
		40-49	.520		0.194		.007		0.140 to 0.901							
		≥ 50	.631		0.215		.003		0.210 to 1.053							
	**Location (reference:** **Northeast Region** **)**												0.005			
		Eastern Region	–.204		0.217		.35		–0.630 to 0.222							
		Central Region	–.379		0.237		.11		–0.843 to 0.086							
		Western Region	–.341		0.242		.16		–0.816 to 0.134							
	Marital status		–.119		0.094		.20		–0.303 to 0.065		0.002					
	**Education level (** **reference:** ***<*** **high school** **)**												0.012			
		High school’s degree	–.189		0.195		.33		–0.572 to 0.193							
		Bachelor’s degree	–.442		0.179		.01		–0.794 to –0.090							
		> Bachelor’s degree	–.432		0.235		.07		–0.893 to 0.029							
	**Annual income (** **reference:** **≤ ¥50,000** **)**												0.010			
		¥50,001-100,000	–.319		0.131		.02		–0.575 to 0.063							
		¥100,001-150,000	–.108		0.146		.46		–0.395 to 0.179							
		¥150,001-200,000	.010		0.186		.96		–0.354 to0.375							
		≥ ¥200,001	–.193		0.196		.33		–0.579 to 0.193							

^a^Model 1: *F*_5,752_=168.8; *P*<.001, *R*^2^=0.526.

^b^Model 2: Δ*F*_7,750_=29.7; *P*<.001; Δ*R*^2^=0.033.

^c^Model 3: Δ*F*_23,734_=6.48; *P*<.001; Δ*R*^2^=0.055.

Model 3 (*R*^2^=0.055; Δ*F*_23,734_=6.48; *P*<.001) revealed that gender, age, education level, and annual income were all significant predictors of patients’ overall satisfaction with Chinese internet hospital service while marital status was not. Specifically, patients with a bachelor’s degree were significantly less satisfied with internet hospitals compared to those with a high school (or below) degree (ie, the reference group; B=–.442; *P*=.01; 95% CI –0.794 to –0.090); and patients with an annual income between ¥50,001 and ¥100,000 were significantly less satisfied with internet hospitals compared to those with an annual income below ¥50,000 (US $ 7019.91; ie, the reference group; B=–.319; *P*=.02; 95% CI –0.575 to –0.063). Most importantly, after controlling for the patient’s demographic characteristics, the effects of the service perspectives (tangibles and empathy), as well as familiarity and willingness remained significant. Overall, the 5 SERVQUAL-C dimensional scores contributed significantly to the explanation of variances in patients’ satisfaction, while the inclusion of familiarity and willingness, as well as the sociodemographic variables, only slightly improved the explanation.

## Discussion

### Principal Findings

This study evaluated the service quality of internet hospitals in China via the Chinese version of SERVQUAL from the perspective of patients, and associated patients’ satisfaction. To summarize, the current service quality of Chinese internet hospitals as perceived by patients did not yet meet their expectations, especially in terms of service responsiveness. Factors associated with patient satisfaction suggested that patient familiarity with and willingness to use internet hospitals cast on their overall satisfaction.

In our research, we found that more than half of the sampled participants had used internet hospitals. However, there is a big gap in the penetration rate of internet hospitals between the young and the older people older than 60 years, which may be related to the older people are not used to the internet, and the psychological resistance caused by the fear of fraud. Our results also demonstrated that education level and annual income played an important role in determining whether patients had used internet hospitals. This finding emphasized the necessity of lowering the threshold and fee of using internet hospitals, which can be achieved by creating more user-friendly web pages, providing more smooth operation, or introducing more favorable policies [16], etc.

Furthermore, there was diversity in the patients’ familiarity and willingness to use internet hospitals. The majority of participants found it hard to evaluate their familiarity with internet hospitals, yet many more expressed interest and willingness to use this service. This could be attributable to the lack of publicity and promotion of internet hospital services so patients were not yet aware of the possible convenience and applicability of Chinese internet hospitals. At the same time, the patients’ stronger willingness also pointed out that internet hospitals’ functions could serve as an important supplement to the current health care system and associated health care service to further improve both the health care service and patients’ experience. It was noteworthy that the COVID-19 pandemic had somehow fastened the development of the internet hospital service scope and quality, especially in China [17]. Therefore, it is expected that patients’ willingness toward internet hospitals might further enhance to the extent that they become more and more familiar with its services.

Our results also indicated that patients’ expectations as regards the quality of the provided services are not met. All of the five quality dimensions have a negative gap between patients’ expectations and perceptions. These results are similar to other studies using the SERVQUAL model in China [18] and other countries such as Greece [19-21]. The greatest gap lies in responsiveness (–0.308) and reliability (–0.276). However, the difference between the two dimensions of expectations and perceptions does not necessarily indicate a low quality of service, but rather that the patient’s requirements are not met, thus leading to patient dissatisfaction. IPA results showed that prompt response, clear feedback pathway, and active feedback handling demonstrate high expectations but low perception levels, which covered three of the four items of responsiveness. Gaps revealed in IPA corroborated patients’ reported reasons for and against using internet hospitals. Specifically, timely responses, as well as diagnosis and treatment accuracy, were the most concerning reasons. Improvement in these perspectives would significantly enhance patients’ willingness toward internet hospitals. Another item that warranted research attention was reliable diagnosis, it was undeniable that the convenience of digital interaction inevitably introduced higher risks of misdiagnosis. Therefore, developers should pay more attention to the importance of user-friendly interfaces and feedback from internet hospital users to promote efficient and effective responses. Furthermore, more policies and regulations ought to be adopted to regulate information security and digital health care processes to minimize the perceived information risk and medical risks.

Results revealed that patient-perceived tangibility, reliability, and empathy positively predicted satisfaction, with increased empathy being particularly effective in improving satisfaction. This may be due to the fact that patients originally have low expectations of empathy, and once a high-quality empathic service is perceived, the sense of surprise that comes with it can substantially increase satisfaction. At the same time, responsiveness, which had a low patient perception score, was unexpectedly not a significant predictor of satisfaction, which may be the result of a large gap between perceived and expected aspects of responsiveness, and the fact that if responsiveness is not currently sufficient for normal service delivery, patients will also see improved responsiveness as a basic requirement that should have been achieved and will not be able to establish a link with satisfaction.

In addition, patients’ familiarity and willingness to use the internet hospital were similarly significantly and positively associated with satisfaction, and the association of these dimensions on satisfaction was not affected by the introduction of demographic variables. Therefore, strengthening the publicity and promotion of internet hospitals among the patient population so that more people can learn to use internet hospitals more conveniently and quickly, as well as increasing the willingness of patients to use internet hospitals when they have a need for medical care, can better improve satisfaction and establish a more amicable and stable digital consultation platform.

### Conclusions

Using the SERVQUAL questionnaire, this study shed light on gaps between the expected and the perceived service quality of Chinese internet hospitals from patients’ perspectives. In the future, internet hospitals should focus more on how to improve reliable diagnosis, prompt response, and active and clear feedback pathways. In this way, internet hospitals could improve patients’ satisfaction and provide superior telemedicine services, and consequently, patient’s willingness to use internet hospitals in need.

## Abbreviations

IPA: important-performance analysis
SERVQUAL: Service Quality Questionnaire

## Data Availability

The datasets analyzed during this study are available from the corresponding author upon reasonable request.
